# Phenological sensitivity and seasonal variability explain climate-driven trends in Mediterranean butterflies

**DOI:** 10.1098/rspb.2022.0251

**Published:** 2022-04-27

**Authors:** Pau Colom, Miquel Ninyerola, Xavier Pons, Anna Traveset, Constantí Stefanescu

**Affiliations:** ^1^ Global Change Research Group, Institut Mediterrani d'Estudis Avançats (IMEDEA-CSIC-UIB), Miquel Marqués 21, 07190 Esporles, Mallorca, Balearic Islands, Spain; ^2^ Grumets Research Group, Departament de Biologia Animal, Biologia Vegetal i Ecologia. Edifici C. Universitat Autònoma de Barcelona, 08193 (Bellaterra, Barcelona), Catalonia, Spain; ^3^ Grumets Research Group, Departament de Geografia. Edifici B, Universitat Autònoma de Barcelona, 08193 (Bellaterra, Barcelona), Catalonia, Spain; ^4^ Natural Sciences Museum of Granollers, Francesc Macià 51, 08402 (Granollers, Barcelona), Catalonia, Spain; ^5^ Centre de Recerca Ecològica i Aplicacions Forestals (CREAF-CSIC-UAB), Universitat Autònoma de Barcelona, 08193 (Cerdanyola de Vallès, Barcelona), Catalonia, Spain

**Keywords:** global warming, insect phenology, population trends, species' life-history traits, host plant specialization, long-term trends

## Abstract

Although climate-driven phenological shifts have been documented for many taxa across the globe, we still lack knowledge of the consequences they have on populations. Here, we used a comprehensive database comprising 553 populations of 51 species of north-western Mediterranean butterflies to investigate the relationship between phenology and population trends in a 26-year period. Phenological trends and sensitivity to climate, along with various species traits, were used to predict abundance trends. Key ecological traits accounted for a general decline of more than half of the species, most of which, surprisingly, did not change their phenology under a climate warming scenario. However, this was related to the regional cooling in a short temporal window that includes late winter and early spring, during which most species concentrate their development. Finally, we demonstrate that phenological sensitivity—but not phenological trends—predicted population trends, and argue that species that best adjust their phenology to inter-annual climate variability are more likely to maintain a synchronization with trophic resources, thereby mitigating possible negative effects of climate change. Our results reflect the importance of assessing not only species' trends over time but also species’ abilities to respond to a changing climate based on their sensitivity to temperature.

## 1. Background

The effects of anthropogenic climate change have been documented for a wide range of taxa [1], of which insects—and, specifically, butterflies—are one of the best-studied groups ever since research into this question began [2,3]. The remarkable sensitivity of these ectothermic organisms to changes in biotic and abiotic conditions, together with the availability of long-term databases covering large geographical areas generated by citizen science projects, have made this group a study model [4]. The ecological literature in recent decades has highlighted how changes in the population size [5–7], distribution [3,8,9] and phenology [5,10,11] of butterflies are being driven by an ever-warmer climate. Nevertheless, how population trends are being affected by these various changes is less clear (but see [12,13]).

The advancement of spring emergence due to increasing temperatures is the most frequently recorded phenological trend in butterflies [5,14,15]. However, intra- and interspecific variation in the degree of this response may vary due to latitudinal [16,17] and seasonal [15,18] climatic variation. In the Mediterranean basin, in particular, there have been strong seasonal differences in the magnitude of warming [19]. Therefore, we would expect the strength of phenological trends to depend on the specific period of the year in which the development of the immature stages is affected by the ambient temperature. However, to date, no study has yet assessed this hypothesis.

Despite the considerable amount of data documenting phenological shifts, little is known about how changes in the timing of emergence influence butterfly population dynamics. Advancing emergence may have several positive implications. First, it can reduce the time the immature stages need to develop, and thus, the time they are exposed to potential predators and parasitoids [20,21]. Second, it may increase the time available to complete generations, with potential benefits for abundance trends [22,23]. Finally, it may ensure good synchronization with the phenology of plants used as nectar sources, which are also expected to advance their flowering due to warming [24]. On the other hand, some studies have suggested that phenological advances could in fact be maladaptive. For instance, they could be detrimental if they lead to the addition of new generations that do not have enough time to enter diapause before the arrival of winter (i.e. developmental trap; [23,25]). Taken together, all these possible positive and negative effects involve great complexity to produce accurate predictions regarding the effects of changing the time of emergence on abundance trends.

Recent research has shown positive effects of phenological advancement on abundance trends in Lepidoptera of North and Central Europe [12,13]. However, phenological effects on abundance have only been investigated using phenological trend as the predictor variable, while none of these studies used phenological sensitivity to climate as a predictor of population dynamics. Here, we explored the idea that species that show high plasticity of phenology to inter-annual climate variability have a greater ability to cope with climate change, as this trait will favour a greater synchronization with trophic resources that can be highly plastic (e.g. [26]), and therefore, it would increase individual fitness. We hypothesize that species with high sensitivity to climate—that is, species more likely to advance their phenology in warmer years but also to delay it in colder years—have experienced more positive or fewer negative abundance trends in recent decades.

## 2. Material and methods

### (a) Study area and butterfly data

The study area lies in the north-west Mediterranean basin (*ca* 32 600 km^2^) in an area in which butterflies have been systematically monitored since 1994 within the framework of the Catalan and Andorran butterfly monitoring schemes (CBMS and BMSAnd; see www.catalanbms.org) (electronic supplementary material, appendix, figure S1).

Butterfly abundance data were recorded weekly from March to September (i.e. *ca* 30 recording events each year depending on the weather conditions) along fixed transects following a standard methodology [27,28]. We used data from a 26-year period (1994–2019) from 59 sampling sites that had been monitored for at least 10 years. For all possible combinations of species, sites and years, we restricted our dataset to cases in which the focal species were recorded during at least five weeks at a site to avoid unreliable flight curves. A generalized additive model (GAM) was then fitted to the site counts to generate the flight curve of a species for the site and year (see below, ‘generation of variables’), but excluded cases in which the model did not converge or failed to find at least one peak of abundance. Finally, we discarded (i) migratory species (i.e. when records mostly correspond to immigrant individuals; e.g. *Vanessa cardui*); (ii) multi-voltine species in which the different generations greatly overlap (e.g. *Pararge aegeria* and *Polyommatus icarus*) and (iii) species for which we had data from less than three populations. Our final dataset included 553 populations of 51 species that were statistically robust enough to estimate long-term trends in both abundances and phenologies.

### (b) Ecological and life-history species traits

We used six traits that could potentially explain trends in abundance and phenology, and differences in species' sensitivity to temperature [11,12,29,30]. Three traits were categorical: voltinism (univoltine versus multi-voltine), overwintering stage (egg, larvae, pupa or adult) and larval diet composition (grass, forb or tree); the other three traits were continuous variables related to adult habitat preference and specialization, and to larval trophic specialization: (i) a habitat species specialization index (SSI) [31]; (ii) an index to estimate butterfly preferences for open or closed habitats (TAO index, [32]) and (iii) the host plant index (HPI, [33]) that quantifies the trophic specialization of a butterfly species in the larval stage based on the number of families, genera and plant species used in the study region. This last variable was normalized using a square-root transformation. See electronic supplementary material, appendix, text S1 for further details of the trait calculations.

### (c) Temperature data

Month-by-month mean air temperature (minimum, mean and maximum) raster surfaces were obtained using a two-step (global and local) spatial interpolation (see [34] for details). First, a multiple linear regression using geographical predictors (altitude, latitude, continentality and solar radiation) was performed for each month and climatic element. Secondly, an inverse distance weighting residual local interpolation was used to correct these surfaces. This method is based on data from *ca* 3500 meteorological stations run by the State Meteorology Agency (AEMET) and a quasi-automatic quality control suite, applied to deal with this massive dataset and to remove the effects of non-climatic factors (i.e. short length series, non-stable or poorly curated data, or station relocation accompanied with geolocation refinements). The obtained maps gave a cross-validated root mean-squared error of *ca* 1°C for all months. Initial 100 m surfaces were densified into 2 m surfaces (using bilinear interpolation) to create new vertices in the butterfly sampling sites. Then, we enriched these vertices with the pixel values from the climate rasters and computed the mean temperatures for each site.

### (d) Abundance and phenological estimates

For each population, we calculated annual abundance and phenological estimates. Annual abundance was calculated as the logarithm of the total number of individuals recorded in a year. We divided the total number of individuals by the number of recording events as in some years, the transects were not sampled during all the 30 weeks due to bad weather conditions in a few weeks. To estimate population phenology, we followed the approach used by Macgregor *et al*. [12] and fitted abundance data using GAM models with a Poisson error distribution with a restricted maximum likelihood. This method is less sensitive to variation in sample sizes and species detectability and provides more accurate estimates than methods based on first appearance dates [35]. We used the date (Julian day) of the first annual emergence peak predicted by the models as the annual phenological estimate for both univoltine and multi-voltine species (see electronic supplementary material, appendix, text S2 for more details and specifications on how these calculations were refined for each species).

### (e) Data analysis

#### (i) Temperature trends

We first analysed the overall temperature trends for our 59-site dataset. We carried out generalized linear mixed models (GLMM) for the mean monthly and annual temperatures (response variables), using year as a predictor and site as a random effect, and assuming a Gaussian error distribution. We repeated the same analysis with maximum and minimum temperatures as response variables. We also calculated the mean annual trends for each site separately with linear regressions. Finally, we tested the spatial variability of our dataset using a categorization of the sites’ climatic region with a three-level factor (alpine and subalpine, Mediterranean mesic and Mediterranean xeric). We performed a generalized linear model (GLM) with the slope of the site trend (i.e. annual mean temperature versus year) as the response variable and the climate region as the predictor variable, again adjusting errors to a Gaussian distribution.

#### (ii) Abundance and phenological trends

Abundance and phenological trends were calculated at both population (*n* = 553) and species (*n* = 51) levels. Population trends were calculated as the slope of a linear regression with abundance (i.e. logarithm of the mean abundance) and phenology (i.e. Julian day) as response variables and year as the predictor variable, with errors adjusted to a Gaussian distribution. Species trends were calculated with the same structure but using GLMMs and controlling for the site factor as a random effect.

#### (iii) Phenological sensitivity

To assess the sensitivity of species phenology to ambient temperature, we used a time-window approach [15]. We calculated the mean temperatures of 36 potential time-windows ranging from one to three months from September of the previous year to September of the current year. We used these potential time-windows to identify the period of the year with the highest temperature effect on the phenology of each species (hereafter, the *critical period*). For each time-window and species, we fitted the phenological data using a GLMM with temperature as a predictor, site as a random effect and a Gaussian error distribution. For each species, we selected the best-fit model according to the Akaike information criteria. We used the slope of the best-fit models to estimate species' phenological sensitivity.

#### (iv) Species trends related to ecological and life-history traits

To test the effects of species traits on phenological sensitivity and trends in abundance and phenology, we used phylogenetic generalized least-squares (PGLS) to control for the potential phylogenetic signal, as we assumed that more closely related species are more likely to have similar ecological and life-history traits [36]. To construct the phylogenetic tree of our species subset, we used recently published data on the phylogeny of European butterflies [37] and PGLS to analyse the effect of six traits (see above, ‘Ecological and life-history species traits’) on (i) phenological sensitivity, (ii) phenological trends and (iii) abundance trends.

#### (v) Relationship between abundance trends and phenological variables

We performed several PGLS models to test to what extent phenology can explain abundance trends. First, we tested whether greater phenological sensitivity results in a steeper phenological trend. For this, we excluded three species as outliers because they have positive values of phenological sensitivity (i.e. responded with phenological delays to an increase in temperature; see electronic supplementary material, appendix, text S3) in contrast with the other 48 species with negative values (i.e. responded with expected phenological advancements to an increase in temperature) (figure 2*c*). We used the absolute value of the slope of the trend over time (hereafter, the *absolute phenological trend*), which quantifies the degree of change without considering its direction (i.e. either advancement or delay). This variable was first normalized with a square-root transformation because of its skewed distribution. Finally, we tested the effect of phenological sensitivity, phenological trend and absolute phenological trend on abundance trends. We used both the real and absolute values of the trends as variables to test separately the effect of the direction and the magnitude, respectively, of the changes in the emergence peak on the abundance trends. We first ran the models including only a single predictor variable (either phenological sensitivity, phenological trend or absolute phenological trend) and then also included the significant species traits related to abundance trends.

All analyses were carried out using R v. 4.1.1 [38]. The following packages were used: *mgcv* [39] to fit GAM models, *lme4* [40] to conduct GLMMs, *ape* [41] to carry out the PGLS analyses, and *MuMIn* [42] for model comparison.

## 3. Results

### (a) Trends in local temperature

**Figure 1.**
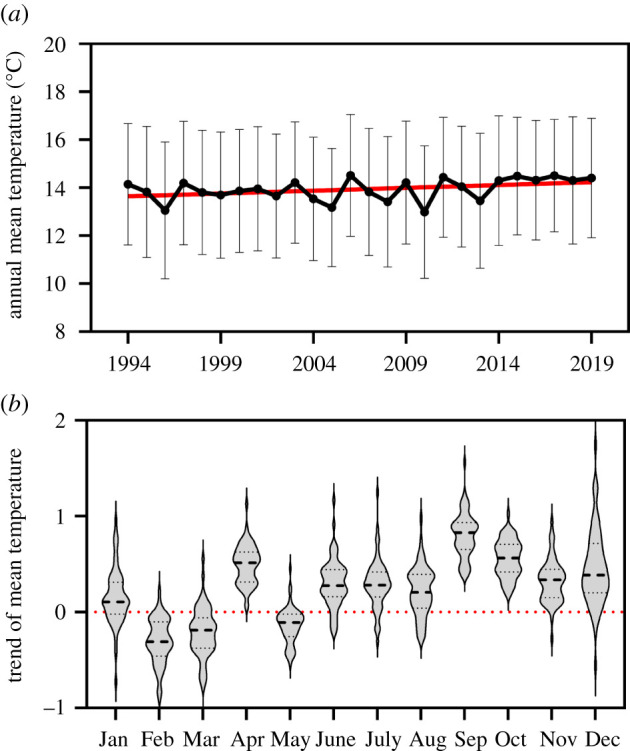
Annual and monthly mean temperature trends over a 26-year period (1994–2019). (*a*) Positive significant trend in annual mean temperature. The red line shows the linear regression line over time. Bars represent the standard deviation between the 59 study sites. (*b*) Violin plots show the frequency distribution of the slopes of the linear models between temperature and year for the 59 study sites in each month. The black dashed line in each violin plots represents the median of the distribution. The red dotted line at the zero value on the *y*-axis is shown to distinguish between negative and positive values. (Online version in colour.)

Our models estimated an increase of 0.59°C in mean temperatures (*t* = 14.02, *p* < 0.01) (figure 1), 0.55°C in maximum temperatures (*t* = 8.64, *p* < 0.01) and 0.42°C in minimum temperatures (*t* = 7.18, *p* *<* 0.01), on average, for the 59 sites during the 26-year period (1994–2019) (electronic supplementary material, appendix, figure S2). According to individual linear models, 51% of sites showed a significant increase in mean temperature but no site showed a significant decrease in this variable (electronic supplementary material, appendix, table S1). Nevertheless, monthly temperature trends for all sites showed remarkable seasonal variability. Mean temperatures increased significantly in nine months of the year but decreased significantly in February, March and May (figure 1; electronic supplementary material, appendix, table S2). A similar seasonal pattern was observed when we used an independent dataset of average temperatures recorded in a network of 24 automatic weather stations for the entire study region (Meteorological Service of Catalonia, data not shown). No differences in temperature trends were observed between climatic regions, which allowed us to assume that spatial variability is not significant in our dataset.

### (b) Trends in abundance and phenology

**Figure 2.**
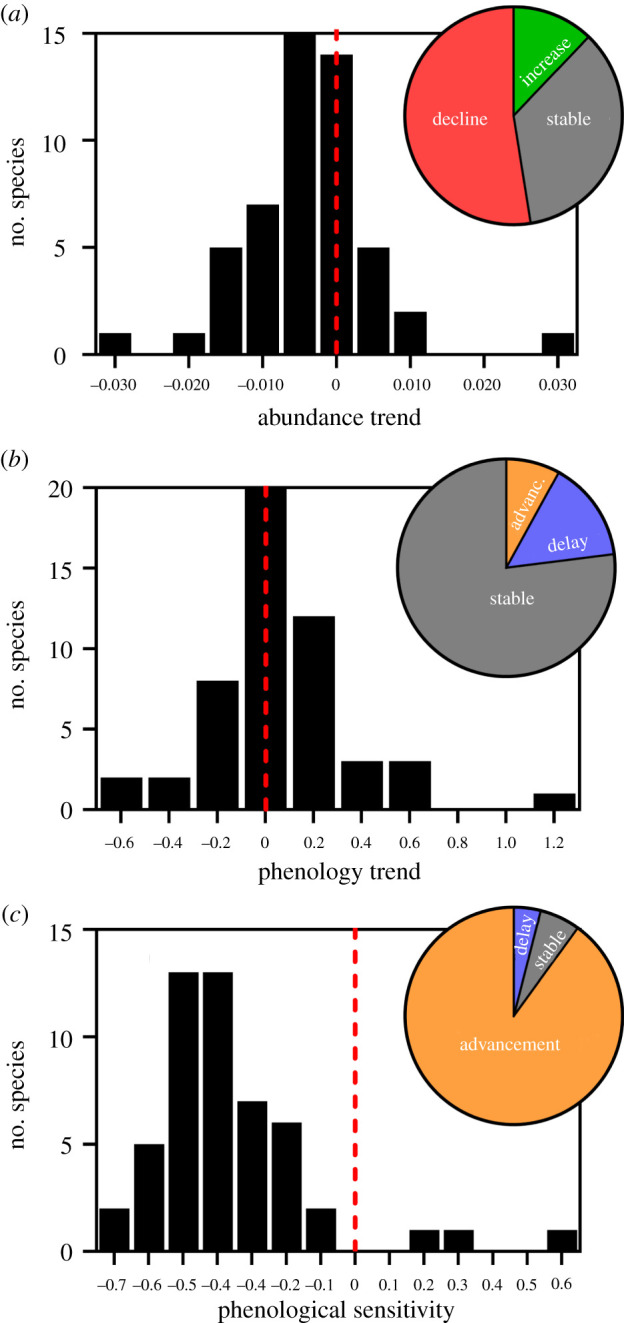
Abundance, phenology and sensitivity estimates for the 51 studied butterfly species. The estimates are the slopes of the GLMMs with (*a*) the logarithm of mean abundance as response variable and year as predictor; (*b*) Julian day of the emergence peak as response variable and year as predictor; (*c*) Julian day of the emergence peak as response variable and mean temperature of the critical period as predictor. The dashed line at the zero value on the *x*-axis is shown to distinguish between negative and positive values. Negative values of phenological trends and phenological sensitivity indicate advances in butterfly emergence in time or responses to increasing temperature, respectively, while positive values indicate delays in butterfly emergence. Pie charts show the percentage of species in each category. (Online version in colour.)

Of the 553 analysed populations, 33% showed significant trends in abundance, of which 75% were negative (i.e. populations declined during the study period). Similar results were observed at species level, with a tendency for abundances to decline (figure 2*a*); 53% of species significantly declined in abundance, while only 12% showed significant positive trends (electronic supplementary material, appendix, table S3). Regarding phenology, only 3% of the populations advanced their emergence peaks, while 3% delayed them. At species level, 8% and 15% of species advanced and delayed their peaks, respectively (figure 2*b*; electronic supplementary material, appendix, table S3).

### (c) Butterfly sensitivity to climate

**Figure 3.**
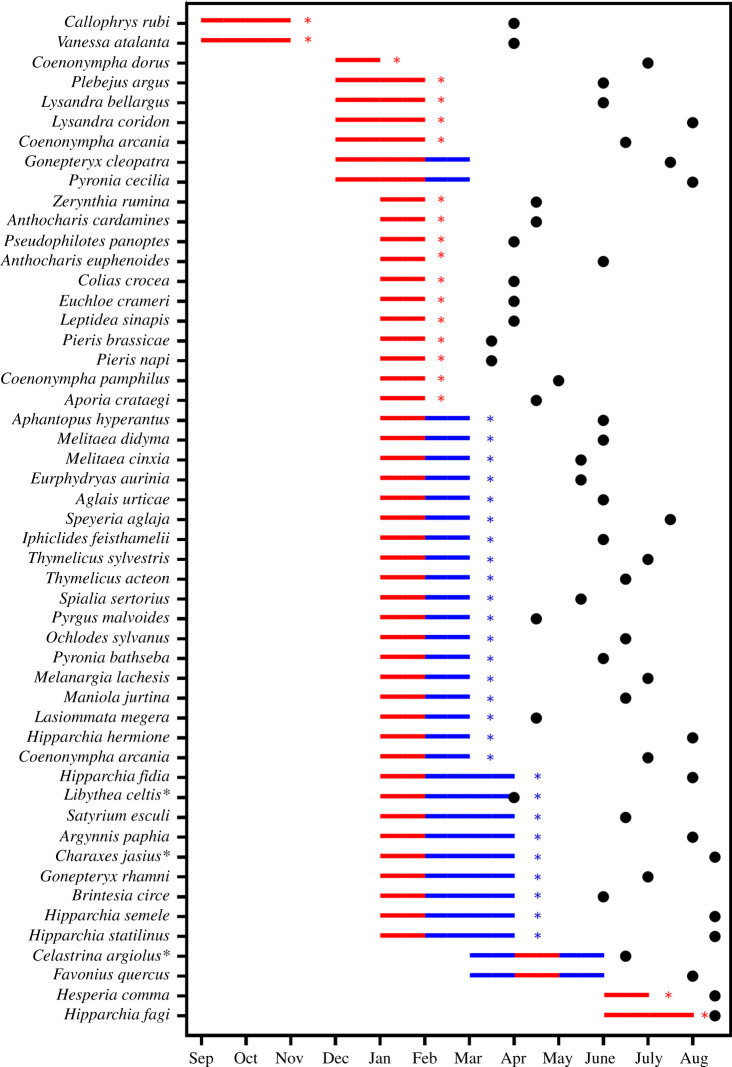
Species’ critical periods. The period from September of the previous year to September of the current year in which the sensitivity (i.e. the relationship between the temperature and the emergence peak) is greatest. Lines represent the critical period of each species: in red, months with positive trends in temperature; in blue, months with negative trends in temperature (figure 1; electronic supplementary material, appendix, table S2). Red and blue asterisks show respectively positive and negative significant trends of the critical period. Points represent the mean peak day of each species*.* (*) We used the peak inactivation of hibernating adults for *Libythea celtis* and the peak in the second generation for *Charaxes jasius* and *Celastrina argiolus* (electronic supplementary material, appendix, text S2). (Online version in colour.)

The effect of temperature on butterfly emergence peak was negative for most species, indicating a clear trend to advance the flight period as temperatures increase (figure 2*c*). Critical periods varied between species (figure 3). Temperature effects on emergence peaks were greatest in the autumn of the preceding year (for *Callophrys rubi* and *Vanessa atalanta*), in winter (for most species) or in spring–summer (e.g. for *Favonius quercus* and *Hesperia comma*). Up to 86% of the species included January in their critical period, 57% February and 22% March. An increase in the annual temperature of the critical period produced significant advances in 90% of species' phenology (electronic supplementary material, appendix, table S3). Only two species responded to warming with a significant phenological delay (*Favonius quercus* and *Hipparchia fagi*; see electronic supplementary material, appendix, text S3), while three species did not respond significantly to temperature changes (*Libythea celtis*, *Hipparchia semele* and *Spialia sertorius*).

### (d) Interspecific variability in butterfly trends modulated by species traits

**Figure 4.**
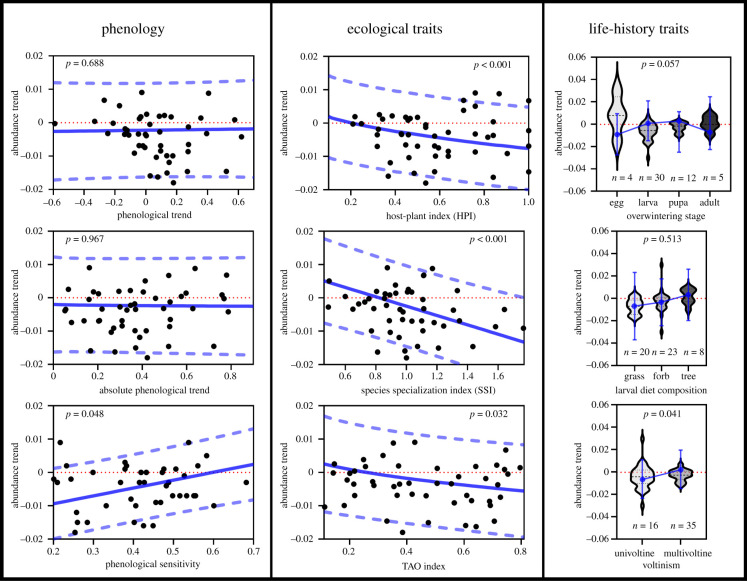
Predictor effect plots for abundance trends from PGLS tests. Black points and violin plots show the distribution of the data. Blue lines and points depict model-predicted relationships between abundance and independent variables. Dashed blue lines and bars indicate 95% confidence intervals. The dashed red line at the zero value on the *y*-axis is shown to distinguish between negative and positive values. (Online version in colour.)

Several traits showed to be significant predictors of abundance trends, including HPI, voltinism, SSI and TAO index (figure 4; electronic supplementary material, appendix, table S4). Species using fewer host plants (i.e. trophic specialists), univoltine species, habitat specialists and species with a preference for more open habitats had the greatest declining trends. However, the model comparison indicated that larval trophic specialization (measured as HPI) was the best predictor explaining abundance trends. Larval trophic specialization was also the only significant predictor of phenological trends, which suggests that specialized species experience a greater degree of phenological delay (electronic supplementary material, appendix, table S4). Species sensitivity was not significantly predicted by any species trait (electronic supplementary material, appendix, table S4).

### (e) Effects of phenological responses to climate on abundance trends

The absolute phenological trend was significantly associated with phenological sensitivity (electronic supplementary material, appendix, table S5). The greater the sensitivity to temperature a species had, the greater change in the emergence timing experienced (phenology delay or advancement) during the study period. Phenological sensitivity also significantly predicted abundance trends, both as the sole predictor and when all significant species traits (electronic supplementary material, appendix, table S5) were included in the model. Species with greater sensitivity to temperature experienced more positive or fewer negative abundance trends (figure 4). Neither the absolute phenological trend nor the real phenological trend variables had any significant effect on abundance trends, regardless of including the significant species traits in the model.

## 4. Discussion

### (a) Phenological trends

Overall, our findings contrast with the expected pattern of phenological advancement under a global warming scenario [1,5]. Indeed, recent work based on similar data and lengths of time series to our study supports the idea that in recent decades, phenological advancements may not have been as common as predicted [13]. Only 8% of the species in our dataset underwent significant advances and, surprisingly, 15% of the species in fact delayed their phenology. Reports of phenological delays often contain sampling errors (i.e. short time series; [43]). However, this does not seem to be the case here because we used time series with an average length of 24.9 years for our calculations. Although mean temperatures in the region increased by 0.59°C, the decrease in mean temperatures in a few particular months could be key to explain the variability in species’ phenological trends. In fact, our results are consistent with the seasonal variability of climate trends described for the Mediterranean region, with a greater contribution to warming in summer and autumn than in winter and early spring [19,44]. The observed negative trends of mean temperatures in February, March and May could offset the general warming trend and also explain the delays in some species, as these months are part of the critical period of development in the immature stages of most species. As expected, the most sensitive species to temperature were those experiencing the steeper phenological trends in absolute terms, thereby confirming the hypothesis that butterfly phenology is driven mainly by climate. Interestingly, species with greater trophic specialization in their larval stages delayed their phenology more than generalists. We have no explanation for this pattern, as these specialists depend on very different types of plants (e.g. grasses, forbs and trees) and include species with a wide range of emerging times over the season. More work is needed to understand this relationship, which could have conservation implications if increasing delays eventually become maladaptive for these species.

### (b) Abundance trends

Our analyses once again reveal a worrying decline in butterfly populations in the Mediterranean basin [29], which is even more severe than in other regions of Europe [45]. More than half of our study species decreased in abundance, and probably some of the rarest and most specialized species excluded from our analyses are those undergoing the most serious declines. We identified several species traits related to this pattern. Habitat specialists declined more than generalists as they are more affected by changes in land use (e.g. [46]). Specifically, species preferring open habitats had greater negative trends, probably due to land abandonment and vegetation encroachment in habitats [32]. Contrary to results of a previous study [29], we found that univoltine species underwent steeper declines than multi-voltine species, which adds to the current debate in the literature: the greatest declines have been reported for univoltine species [12,47,48] but also for multi-voltine species [29,49]. Interestingly, the best predictor of abundance trends was larval trophic specialization (i.e. HPI index). Larval specialists, like habitat specialists, are usually the most affected by habitat loss and degradation. In addition, larval specialists are the species that are most potentially affected by a loss of phenological synchrony with their host plants due to climate change [50]. Indeed, larval specialists were the group of species delaying most, or advancing the least, their phenology, which could have led to a greater mismatch with their host plants.

### (c) Effects of phenological shifts on abundance

Abundance trends were found to be associated with phenological sensitivity, but not to phenological trends, confirming the hypothesis that at least for Mediterranean butterflies, phenological sensitivity is a better predictor than phenological trends *per se* for abundance trends. The effect of phenological sensitivity on abundance trends is robust since it remained significant when we controlled for the phylogenetic signal and for the effect of the ecological and life-history traits intrinsically related to abundance trends. In the UK, Macgregor *et al*. [12] found a positive effect for phenological advancement on abundance trends, but as in our work, this effect has not been found in other regions [13,30]. The effect in the UK was only found for multi-voltine species, so the relatively small number of multi-voltine species in our study dataset after removing the multi-voltine species with overlapping generations (*n* = 16) in comparison to the dataset of Macgregor *et al*. [12] (*n* = 39) could have prevented us finding the same pattern. However, multi-voltine species have a greater sensitivity to increase their flight duration than univoltine species [30], and therefore, this pattern could be more related to the positive effects of lengthening the flight period [49] and the increases in voltinism described in northern latitudes (e.g. [22]). Other strategies not analysed in our study, including a northward shift in range boundaries, could also be associated with positive abundance trends [13], although this particular strategy will always be highly dependent on suitable habitat availability [51], and it is possible that only species that already have stable or positive trends could actually successfully undertake range expansions [52].

Although it is often argued that phenological sensitivity to changing climatic conditions may increase or maintain population abundance, this has rarely been tested [53]. Our results actually suggest that phenological sensitivity could be an adaptation to the inter-annual variation of weather conditions in order to maintain synchrony with resources, which have been shown to respond to the increase of temperatures by advancing their phenology ([26,54] and references therein). This could explain why less sensitive species had sharper declines than highly sensitive species, and why they could potentially be the most negatively affected in the current context of climate change. Furthermore, the positive relationship between the degree of larval trophic specialization and population trends could also give indirect support to this hypothesis, as it is the more generalist species that can find resources with different phenologies and therefore minimize the problems arising from the loss of synchrony with these resources (see, e.g. [24]). However, although these explanations seem plausible, further empirical work is needed to investigate whether phenological plasticity actually increases butterfly synchrony with larval host plants and adult nectar plants. Further research will also be needed to identify the traits explaining phenological sensitivity and thus facilitate the identification of vulnerable species to climate change.

Theoretically, climate-driven changes in phenology can have both positive and negative demographic consequences. Shifting phenology in response to climate can be maladaptive under some circumstances (e.g. [25]), but on the other hand, in many cases, it will improve the performance of individuals by increasing the synchronization with other trophic levels. Here, we show that greater phenological sensitivity can prevent population decline in butterflies and, probably, in other ectotherm organisms. Given that phenological climate-driven trends have been documented in many taxa across a high variety of ecosystems and habitats, it is essential to better understand their demographic consequences. We call for further research to disentangle the ecological consequences of phenological climate-driven trends, assessing not only species' trends over time but also species’ sensitivity to temperature and their ability to respond to a changing climate.

## Data Availability

Data are available for the reviewers and editors in the electronic supplementary material [55] and at the following link: https://doi.org/10.6084/m9.figshare.19137512.
